# A Flexible Pressure Sensor with a Mesh Structure Formed by Lost Hair for Human Epidermal Pulse Wave Monitoring

**DOI:** 10.3390/s23010045

**Published:** 2022-12-21

**Authors:** Xue Wang, Zhiping Feng, Peng Li, Luna Wang, Liang Chen, Yufen Wu, Jin Yang

**Affiliations:** 1 Department of Optoelectronic Engineering, Key Laboratory of Optoelectronic Technology and Systems Ministry of Education, Chongqing University, Chongqing 400044, China; 2 Department of Optoelectronic Engineering, Chongqing Key Laboratory of Laser Control & Precision Measurement, Chongqing University, Chongqing 400044, China; 3 College of Physics and Electronic Engineering, Chongqing Normal University, Chongqing 401331, China

**Keywords:** flexible pressure sensor, lost hair, human epidermal pulse wave, pulse wave transmission time

## Abstract

Flexible pressure sensors with the capability of monitoring human vital signs show broad application prospects in personalized healthcare. In this work, a hair-based flexible pressure sensor (HBPS) consisting of lost hair and polymer films was proposed for the continuous monitoring of the human epidermal arterial pulse waveform. A macroscale mesh structure formed by lost hair provides a simplified spacer that endows the triboelectric-based flexible pressure sensor with sufficient contact–separation space. Based on this mesh structure design, the hair-based flexible pressure sensor can respond to the slight pressure change caused by an object with 5 mg weight and hold a stable output voltage under 1–30 Hz external pressure excitation. Additionally, the hair-based flexible pressure sensor showed great sensitivity (0.9 V/kPa) and decent stability after 4500 cycles of operation. Given these compelling features, the HBPS can successfully measure the human epidermal arterial pulses with obvious details at different arteries. The proposed HBPS can also be used to monitor the pulse signals of different subjects. Furthermore, the three different pulse wave transmission time (PTT) values (PTT-foot, PTT-middle, and PTT-peak) can be obtained by simultaneously monitoring human pulse and electrocardiogram signals, which has enormous application potential for assessing cardiovascular system health.

## 1. Introduction

Statistical data from the World Health Organization show that more people die from cardiovascular diseases (CVDs) than from any other cause each year, and more than three-quarters of stroke-related deaths and heart disease occur in low- and middle-income countries. CVDs, which includes myocardial infarction, stroke, hypertension, and coronary artery disease, has become a serious public health problem and is one of the major threats to human health globally [1]. It often occurs suddenly and without any obvious prior symptoms; furthermore, it is difficult for the cardiovascular system to recover its pre-event health state after the first index event, even with effective treatment. Many reports have indicated that long-term physiological signal monitoring is a critical and indispensable method for assessing a patient’s cardiovascular health state and that it provides an opportunity to reduce the progression of CVDs events or organ damage in CVDs patients [2].

The human epidermal pulse wave generated by the periodic contraction and relaxation of the heart is the most representative vital sign [3]. Owing to the fact that variations in the human pulse wave can reflect many physiological and pathological phenomena of the human body, it has historically been widely used in clinical examinations, especially in traditional Chinese medicine [4]. Real-time monitoring of human epidermal pulse waves with detailed information is an effective method of evaluating cardiovascular system health, which is of great significance for achieving the early diagnosis and prevention of CVDs and reducing the risk of CVDs events [5,6].

In recent decades, many wearable technologies and methodologies have garnered substantial interest in pulse wave monitoring due to their flexibility, user-friendliness, and comfort. From the working principle perspective, these technologies can be mainly divided into ultrasonic [7,8], photoplethysmographic [9,10], piezoelectric [11,12,13,14], piezoresistive [15,16], capacitive [17,18,19], and triboelectric [20,21,22,23,24,25,26,27,28] technologies, which have the remarkable capability of capturing high-quality human epidermal pulse waves. Generally, microstructures are introduced into these technologies to improve the performance of capturing pulse signals with obvious details such as advancing wave peak (P_1_), reflected wave peak (P_2_), and dicrotic wave peak (P_3_). This requires additional complicated processing procedures for fabricating wearable sensors, such as photolithography, oxygen plasma etching, and chemical methods, which increase costs and reduce production efficiency. Therefore, developing a wearable, low-cost, high-performance sensor that is easy to make is urgent for high-quality human pulse wave monitoring.

The tempo of people’s lives has changed significantly with the rapid development of society, and hair loss caused by changes in eating habits, excessive use of computers, increased life pressure, malnutrition, and other reasons is also more common [29,30]. Human hair has a diameter of 60–90 μm and a tensile strength of 40–60%, which makes it conducive to becoming a microscale microstructure. Additionally, existing waste sorting methods and the improvement of awareness about waste sorting make it possible to acquire lost hair at a very low cost. If lost hair could be applied to the fabrication of microstructures in a flexible sensor, a low-cost and simple fabrication method of microstructures that does not rely on complex production equipment and processes would be developed. This will not only turn “trash into treasure” but will also improve the performance of flexible sensors for human pulse wave monitoring.

In this paper, we propose a flexible pressure sensor conducted by lost hair and polymer films for use in the continuous monitoring of human epidermal arterial pulse waveforms. The mesh structure formed by the lost hair provides an important and low-cost spacer for the triboelectric-based pressure sensor, which results in an effective contact separation space between the tribo-pair. The fabrication of the hair-based sensor does not require complex equipment but also finds sustainable use for lost hair as a means of enabling human pulse wave monitoring. Due to the hair-based mesh structure design, the flexible pressure sensor shows obvious output signals, even under the stimulation of an object with 5 mg weight, which is conducive to use as a pressure sensor for capturing the details of the human pulse wave. Additionally, the pressure sensor holds a sensitivity of 0.9 V/kPa and demonstrates a remarkable low-frequency response (1–30 Hz). There was almost no degradation in 4500 cycles of continuous operation. Based on these compelling features, the fabricated pressure sensor excels at human pulse wave monitoring on different body parts and on different subjects. Additionally, the pulse wave transmission time can be obtained successfully by simultaneously collecting electrocardiogram signal pulse waveforms. This work demonstrates a hair-based pressure sensor that provides a simple, low-cost, and convenient platform technology with applications in cardiovascular disease prevention and diagnosis.

## 2. Materials and Methods

### 2.1. Structure and Fabrication of the Proposed HBPS

**Figure 1:**
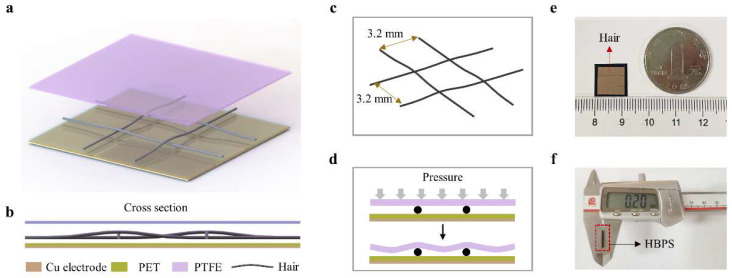
Structure of the designed flexible pressure sensor: (**a**) three-dimensional structure of the proposed hair-based pressure sensor (HBPS), (**b**) A cross section of the HBPS, (**c**) detailed mesh structure formed by lost hair, (**d**) schematic diagram of HBPS deformation under pressure, (**e**–**f**) optical image of the HBPS with a dimension of 12 × 12 × 0.2 mm^3^.

The three-dimensional structure of the proposed hair-based pressure sensor (HBPS), which has a three-layer configuration, is shown schematically in Figure 1a. On the bottom, the polyethylene terephthalate (PET, thickness of 10 μm) film acted as one of the electrification layers, and the Cu layer (thickness of 2 μm) formed by magnetron sputtering on the back of the PET film was used as the conductive layer. For the middle layer, the mesh structure composed of lost hair served as the supporting layer and created a constant contact–separation space between the PET and polytetrafluoroetylene (PTFE, thickness of 20 μm) films. On top, a PTFE film was utilized as another electrical layer. A cross-sectional view shows the multilayer structure of the HBPS, as shown in Figure 1b. Notably, four cross points were formed by mutually perpendicular strands of lost hair; the relative height at these cross points was higher than that of the other areas without a cross point. This created an initial distance between the electrification layers, which provided spacing through a mesh structure and generated effective contact separation. In the fabrication of the HBPS, the electrical wiring is connected with the Cu electrode for outputting electrical signals.

The detailed mesh structure formed by lost hair, which is the core component of the proposed HBPS, is shown in Figure 1c. In two vertical directions, the distance between the two hairs was 3.2 mm. If the distance between the two hairs is too small, the PTFE film cannot effectively contact the PET film when the HBPS is stimulated by external pressure, which results in poor output performance. However, the role of the mesh structure formed by lost hair as a support layer would be weakened if the distance between the two hairs was too large. In this situation, the proposed HBPS could easily reach the saturation state under external pressure. As shown in Figure 1d, The PTFE film would develop a corresponding bending deformation when the HBPS is stimulated by external pressure, which would cause contact–separation between the tribo-pair. The electrical signals will then be generated. Figure 1e,f are optical images of the fabricated HBPS with a flexible, wearable dimension of 12 × 12 × 0.2 mm^3^.

The fabrication procedures of the HBPS are described as follows: first, the PET film was cut with a Cu electrode into 10 × 10 mm^2^. The PET film was pasted flat onto the middle of the biaxially oriented polypropylene (BOPP) film with dimensions of 1.8 × 1.8 cm^2^, and the four sides of the BOPP film were exposed to air. Second, the viscosity of BOPP was used to fix the hair-based mesh structure around it. Third, ultrathin double-sided adhesive tape was applied around the PET film to further fix the hair-based mesh structure. Fourth, The PTFE film is covered on the mesh structure formed by lost hair and fixed with the double-sided adhesive tape in the last step. Finally, the redundant sample was cut off according to the design size.

### 2.2. HBPS Output Performance Characterization

**Figure 2:**
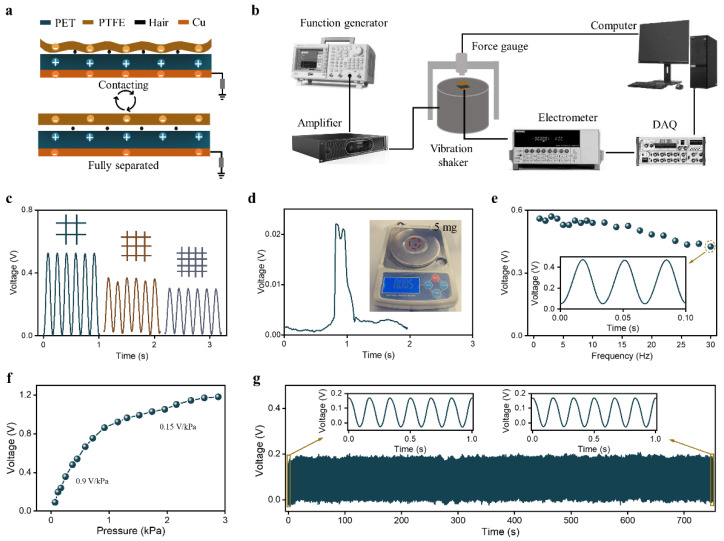
Electrical output and mechanical robustness measurements of HBPS: (**a**) working mechanism of HBPS, (**b**) self-designed testing system for acquiring a quantitative understanding of the electrical output performance of HBPS, (**c**) the output voltage of the HBPS composed of hair with different distance (3.2 mm, 2.4 mm, 1.9 mm), (**d**) output voltage of the HBPS under the pressure of a plastic sheet with 5 mg weight, (**e**) output voltage of the HBPS under a pressure of 0.45 kPa at frequencies ranging from 1–30 Hz, (**f**) pressure sensitivity of the HBPS, and (**g**) output stability of the HBPS.

The working principle of the proposed HBPS, which is based on electrostatic induction and the triboelectric effect, is shown in Figure 2a. In the original state, the PTFE film was separated from the PET film due to the hair-based mesh structure, and there were no output signals. The distance between the PTFE film and the PET film was shortened when external pressure forced the PTFE film close to the PET film. In this process, the effective contact area between the PTFE and PET films gradually increased. Due to the difference in the materials’ tendencies to gain and lose electrons (PTFE film tends to gain electrons, while PET tends to lose electrons), equal amounts of charges with opposite polarity were generated on the surface of the PTFE and PET films. The PTFE film returned to its initial state when external pressure was released, which resulted in electrons flowing from the reference ground to the copper electrode and the production of output signals. The electrons flowed from the electrode to the reference ground when the PTFE film was approached again by the PET film, which generated reversed output signals. With this working mechanism, periodic external pressure signals were converted into periodic electrical signals.

To quantitatively evaluate the output performance of the proposed HBPS, a testing system was developed (see Figure 2b). It consists of a function generator (Beaverton, OR, USA, Tektronix, AFG 3021B), a power amplifier (Suzhou, China, Dongling Vibration Test Instrument Co., Ltd., LabworkPa-13), a vibration shaker (Dongling Vibration Test Instrument Co., Ltd., ET-015), an electrometer (Tektronix, Keithley 6514), a data acquisition card (Austin, TX, USA, National instruments, BNC-2120), a force gauge (Guangzhou, China, SIMBATOUCH, SBT630), and a LabVIEW (National instruments, LabVIEW 2017). Specifically, a function generator, power amplifier, and vibration shaker were used to generate standard excitation signals, and a force gauge was used to capture the pressure of the excitation signal. An electrometer, data acquisition card, and computer were used to obtain the output waveforms of the HBPS. In order to investigate the influence of the distance between the adjacent hairs on the sensor performance, we have made three HBPSs composed of hair with different distances (3.2 mm, 2.4 mm, 1.9 mm). As shown in Figure 2c, it can be found that the output voltage amplitude of the HBPS under the same pressure (0.45 kPa) decreases with the reduced distance between two adjacent hairs. This is because the shorter distance makes it more difficult for the PTFE film to make full contact with PET, resulting in a reduction in the output voltage. To investigate the capability of the proposed HBPS’s response to slight pressure, we tested the output signal of the proposed HBPS under the excitation of a plastic sheet with a 5 mg weight. An obvious 0.022 V voltage is shown in Figure 2d, which confirms the remarkable ability of HBPS to capture tiny pressure signals. In addition, we measured the output voltage of the HBPS under a pressure of 0.45 kPa with various frequencies ranging from 1–30 Hz, as shown in Figure 2e. When the frequency of the excitation signal is greater than 16 Hz, the output voltage of the pressure sensor decreases with the increasing frequency. The illustration shows that even under the excitation of pressure with a frequency of 30 Hz, there is no distortion in the output waveforms.

Sensitivity is an important parameter for evaluating the output performance of a flexible pressure sensor, which is defined as the ratio of voltage change to pressure change. As shown in Figure 2e, the sensitivity curve of HBPS showed two different trends. In the low-pressure range (<0.7 kPa), the HBPS showed a sensitivity of 0.9 V/kPa. The sensitivity decreased to 0.15 V/kPa when the pressure ranged from 0.7 to 2.8 kPa. This is attributable to the difference in the effective contact separation area under different pressures. Additionally, output stability plays an important role in the long-term monitoring of physiological signals. Therefore, the output voltage of the HBPS was continuously measured under the same pressure (0.2 kPa, 6 Hz). As shown in Figure 2f, the output voltage remained stable in 4500 loading/unloading cycles, indicating that HBPS has practical applications.

### 2.3. Human Epidermal Pulse Wave Monitoring

**Figure 3:**
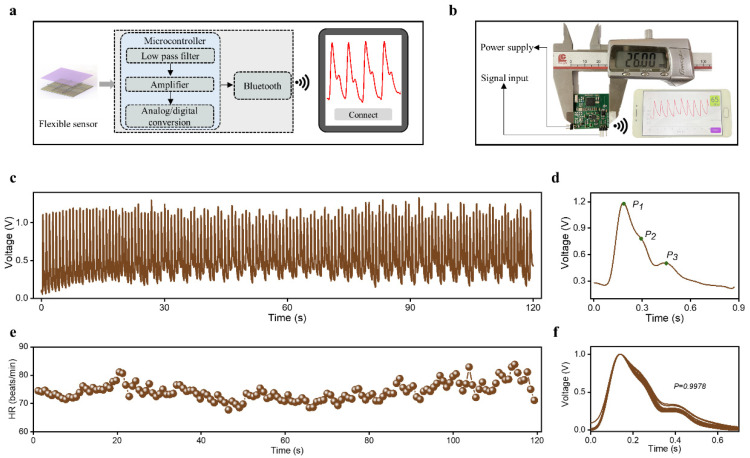
The developed monitoring system and human pulse wave monitoring: (**a**) schematic diagram of the pulse wave monitoring system, (**b**) optical images of the hardware circuit and mobile phone application terminal, (**c**) measured human pulse wave at wrist in 2 min, (**d**) one cycle of the pulse wave, (**e**) real-time HR calculated from the pulse wave, and (**f**) consistency of pulse signals in different cycles.

To make the measurement process more convenient, we built a miniaturized wireless system (Figure 3a). The system consists of three parts: the HBPS for acquiring raw pulse wave signals, the hardware circuit for signal preprocessing, and the mobile phone application terminal for real-time display. The hardware circuit includes a low-pass filter, an amplifier, an analog-to-digital conversion, and a Bluetooth transmission module. The optical images of the hardware circuit with dimensions of 2.7 × 2.6 × 0.5 cm^3^ and the mobile phone application terminal, which provides a convenient and reliable platform for pulse wave monitoring, are illustrated in Figure 3b.

Due to its great output performance, HBPS exhibits promising potential in monitoring human epidermal pulse waves. As shown in Figure 3c, the pulse wave of a 26-year-old subject was successfully measured at the wrist. From the enlarged view of one cycle (Figure 3d), three obvious peaks are observable: P_1_, P_2_, and P_3_. These feature peaks are jointly generated by cardiac ejection and the reflection of tissue structure on blood, which is critical for assessing the cardiovascular system, including vascular elasticity, peripheral resistance, and degree of arteriosclerosis. Heart rate (HR) is a vital sign that indicates the state of the cardiovascular system. For a group of pulse wave signals over a period of 2 min, the real-time HR value was calculated by seeking the local maximum within a 0.6-s interval. As shown in Figure 3e, the calculated HR did not change dramatically and showed an average of 73 beats/min, indicating that the subject’s physiological condition was in a stable state for a short period of time. Of course, there were some small fluctuations in HR that were consistent with human physiological characteristics. The average correlation coefficient between the different periodic waveforms was as high as 0.9978, which indicates that the proposed HBPS has a remarkable ability to stably monitor human pulse signals.

**Figure 4:**
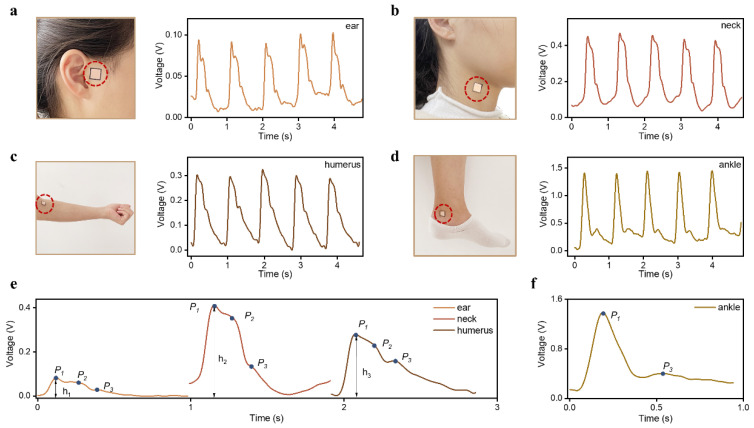
Human pulse wave monitoring at different body parts: (**a**) ear, (**b**) neck, (**c**) humerus, and (**d**) ankle; (**e**) pulse wave at different parts of the human body over one cycle; and (**f**) one cycle of pulse wave measured at the ankle.

As many are aware, blood vessels are located throughout the human body, and it is possible to feel one’s pulse beating by placing a finger on different arteries [31]. The pulse wave at these different arteries plays an important role in the evaluation of vascular elasticity and arteriosclerosis [32]. To verify that the proposed HBPS can be applied to different arterial parts, we attached the HBPS directly to four different arteries to capture pulse signals, including the ear, neck, humerus, and ankle. As shown in Figure 4a–d, the pulse waveforms at these four positions were obtained successfully. The pulse waves at different parts all showed the waveform shape of a typical pulse signal, including an ascending branch and a descending branch, with obvious characteristic peaks on the descending branches. Pulse waveforms of one cycle at different arterial positions are presented in Figure 4e,f. From an amplitude point of view, the amplitude of the pulse wave at the ankle was the strongest, followed by the neck. The ear was the weakest, which is a normal physiological phenomenon related to different factors, such as blood vessel pathways, tissue thickness, and bone structure. The three groups of upper limb pulse waveforms all showed three obvious feature points (P_1_, P_2_, and P_3_), but only two peaks (P_1_ and P_2_) were shown in the pulse signal at the ankle. This phenomenon has been reported in other studies [7,13] and might be caused by the superposition of the advancing wave and the reflected wave. These results indicate that the proposed HBPS is capable of monitoring pulse waves at most arterial positions.

**Figure 5:**
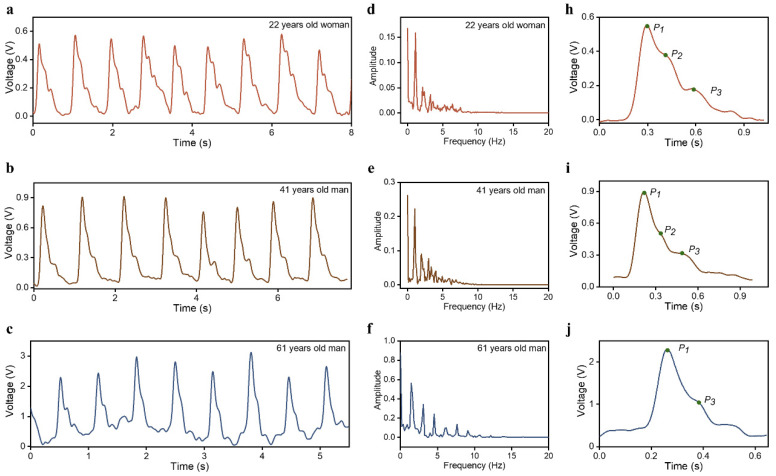
The pulse waves of different subjects: (**a**–**c**) a 22-year-old woman, a 41-year-old man, and a 61-year-old man; (**d**–**f**) the spectrum of pulse signals of different subjects; and (**h**–**j**) an enlarged view of one cycle of pulse waves of different subjects.

In the process of pulse signal analysis, the relationship between the pulse signals of different subjects and cardiovascular disease parameters is often investigated. Therefore, for practical applications, the proposed HBPS’s use in pulse wave monitoring must provide accurate measurements for different individuals. Figure 5 presents the results of the measurement using the same HBPS on three volunteers: a healthy 22-year-old woman (Figure 5a), a healthy 41-year-old man (Figure 5b), and a healthy 61-year-old man (Figure 5c). The frequency spectrums of the pulse signals of the three volunteers are shown in Figure 5d–f. Their main frequency components were concentrated below 10 Hz. For the younger individuals, three feature peaks existed and were obvious, but there was no clear peak P_2_ in the pulse signal of the older volunteer. This is likely due to the fact that the stiffness of arteries increases with age, which eventually leads to the early return of the reflected wave or the superposition of the advancing wave. The obvious two peaks (P_1_ and P_2_) show that the proposed HBPS is capable of capturing the details of pulse signals, even for the elderly, which is of great significance for the evaluation of CVDs, which often occur in middle-aged and elderly populations.

### 2.4. PTT Measurement

**Figure 6:**
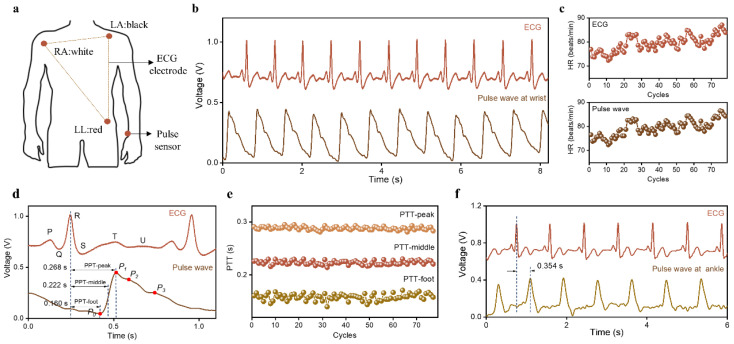
Measurement of PTT: (**a**) schematic diagram of ECG electrode position, (**b**) ECG signals and pulse waves of the same individual obtained by the ECG electrode and the HBPS, (**c**) HR obtained from ECG and pulse wave, (**d**) definition of PTT, (**e**) changing trend of PTT over 78 consecutive cycles, and (**f**) ECG and pulse wave measured at the ankle.

Pulse transit time (PTT), the time taken for the human epidermal pulse wave to travel from the heart to the peripheral arterial position, is a potential indicator for the assessment of CVDs in medical applications [33]. The relationship between PTT and cardiovascular system status has been investigated by many researchers [34]. PTT changes regularly with changes in vascular wall elasticity, peripheral resistance, and blood viscosity [35]. Therefore, PTT-based early prevention and diagnosis of CVD has received considerable attention due to its noninvasive and comfortable measurement form [36,37]. In this work, PTT was obtained using HBPS and an electrocardiogram (ECG) lead-III. The position of the ECG electrode is shown in Figure 6a. The left arm electrode (LA) was placed where the left upper limb connected to the torso. The right arm electrode (RA) was placed where the right upper limb connected to the trunk. The left leg electrode (LL) was attached to the left hip. These three ECG electrodes were used for ECG signal acquisition. The proposed HBPS was attached to the subject’s radial artery to capture the pulse wave signals. Before measurement, the volunteer was required to remain in a resting state for 5 min. During the measurement, the volunteer was asked to maintain a comfortable sitting posture, with legs uncrossed and feet flat on the floor. The brachial artery remained at the level of the left ventricle, and the volunteer was asked to avoid talking and to also relax during the measurement. The ECG signal and pulse wave were sampled at 500 Hz via a data acquisition card (National instruments, BNC-2120).

Figure 6b presents the measured ECG signals and pulse waveforms in 8 s. All of the signals showed good periodicity and typical waveform morphological characteristics. The real-time HR value was calculated by seeking the local maximum within a 0.6-s interval, as shown in Figure 6c. These two groups of signals showed the same HR value in the same cycle and exhibited completely consistent heart rate changes over 78 consecutive cycles; the ECG and pulse signals of the same cycle came from the same cardiac cycle. Figure 6d shows the ECG and pulse wave during one cycle and exhibits five distinguishable peak points, including the P, Q, R, S, T, and U peaks. Three peaks (P_1_, P_2_, and P_3_) were clearly displayed in the pulse waves measured at the wrist. The definitions of the three PPTs (PTT-foot, PTT-middle, and PTT-peak) are also illustrated in Figure 6d. These refer to the time difference between the R peak of the ECG signal and the P_0_ point of the pulse signal, the time difference between the R peak of the ECG signal and the midpoint of the rising branch of the pulse signal, and the time difference between the R peak of the ECG signal and the P_1_ point of the pulse signal. Based on the PTT calculation method, we obtained the change trend of the PTT values over 78 consecutive cycles (Figure 6e). The three PTT values were stable at 0.160 s, 0.222 s, and 0.268 s, respectively, indicating that the physiological state of a healthy individual did not change dramatically in a short time. Moreover, these three kinds of PTTs showed the same change trend, which suggests that the proposed HBPS can monitor physiological signals with accuracy similar to that of a commercial ECG electrode. The ECG signals and pulse waves at the ankle were measured simultaneously (Figure 6f). Compared with the pulse wave at the wrist, the time difference between the advancing wave peak at the ankle and the R peak of the ECG signal was greater (0.354 s) because the artery at the ankle is farther away from the heart; thus, it takes a longer time for the pulse to travel from the heart to the ankle. These results show that the proposed HBPS has remarkable performance in the context of monitoring human epidermal pulse waves and capturing cardiovascular system parameters. Thus, it offers a novel and convenient method for the early prevention and monitoring of CVDs.

## 3. Conclusions

In summary, we developed a flexible pressure sensor for human epidermal pulse wave monitoring, which consists of lost hair and polymer films (PTFE and PET). As the core part of the sensor design, the mesh structure composed of lost hair provided a more effective space for the contact–separation process between the PTFE and PET films. This application of lost hair not only offers sustainable use for waste but can also enhance the performance of flexible pressure sensors. The mesh structure design endows the HBPS with compelling output performance, including excellent responsiveness to an object with 5 mg weight, a remarkable low-frequency response from 1–30 Hz, a pressure sensitivity of 0.9 V/kPa, and decent stability after 4500 cycles of operation. With these properties, the proposed HBPS can successfully measure human epidermal pulse waves at different body parts, including the ear, neck, humerus, wrist, and ankle. Over the same period of continuous measurement, the consistency of the acquired pulse wave of a single individual at different periods was as high as 0.9978. Moreover, the proposed HBPS can be used for pulse wave monitoring of different subjects, including young, middle-aged, and elderly individuals. Moreover, PTT (including PTT-foot, PTT-middle, and PTT-peak) can be easily obtained by simultaneously monitoring human pulse and ECG signals. The proposed HBPS for use in human epidermal pulse wave monitoring demonstrates strong potential for application in CVDs prevention and diagnosis.

## Data Availability

No new data were created or analyzed in this study. Data sharing is not applicable to this article.
